# Shared Skeletal Support in a Coral-Hydroid Symbiosis

**DOI:** 10.1371/journal.pone.0020946

**Published:** 2011-06-14

**Authors:** Olga Pantos, Ove Hoegh-Guldberg

**Affiliations:** 1 School of Biological Sciences, The University of Queensland, St. Lucia, Queensland, Australia; 2 Global Change Institute, The University of Queensland, St. Lucia, Queensland, Australia; Institute of Marine Research, Norway

## Abstract

Hydroids form symbiotic relationships with a range of invertebrate hosts. Where they live with colonial invertebrates such as corals or bryozoans the hydroids may benefit from the physical support and protection of their host's hard exoskeleton, but how they interact with them is unknown. Electron microscopy was used to investigate the physical interactions between the colonial hydroid *Zanclea margaritae* and its reef-building coral host *Acropora muricata*. The hydroid tissues extend below the coral tissue surface sitting in direct contact with the host's skeleton. Although this arrangement provides the hydroid with protective support, it also presents problems of potential interference with the coral's growth processes and exposes the hydroid to overgrowth and smothering. Desmocytes located within the epidermal layer of the hydroid's perisarc-free hydrorhizae fasten it to the coral skeleton. The large apical surface area of the desmocyte and high bifurcation of the distal end within the mesoglea, as well as the clustering of desmocytes suggests that a very strong attachment between the hydroid and the coral skeleton. This is the first study to provide a detailed description of how symbiotic hydroids attach to their host's skeleton, utilising it for physical support. Results suggest that the loss of perisarc, a characteristic commonly associated with symbiosis, allows the hydroid to utilise desmocytes for attachment. The use of these anchoring structures provides a dynamic method of attachment, facilitating detachment from the coral skeleton during extension, thereby avoiding overgrowth and smothering enabling the hydroid to remain within the host colony for prolonged periods of time.

## Introduction

Most colonial hydroids possess an external chitinous perisarc which in athecate species encloses the stolonal hydrorhiza and hydrocaulus, and in thecate individuals extends into a cup-shaped hydrotheca surrounding the hydranth [1]. This chitinous exoskeleton provides the hydroid with structural support and protection from predators. Where hydroids form symbiotic relationships with other organisms, the perisarc may become superfluous and is typically lost, with support and protection being gained from the skeleton of their host. The influence of this type of relationship is particularly evident in members of the genus *Eutima* which includes both free living species that possess a perisarc, and symbiotic species do not [2]. Several *Zanclea* species live symbiotically within the calcareous skeleton of their bryozoan hosts and are able to retract into the host's skeleton, making it redundant for them to produce their own perisarc [3], [4]. The presence or absence of a perisarc providing a physical barrier between the hydroid and its host is also not necessarily indicative of the occurrence of a host response and modification its colony structure. For example, *Ralpharia neira* lives within the tissues of its octocoral host and has a skeletal axis that envelops the perisarc tube forming a gall [5], where as *Zanclea divergens*, has a perisarc-free colony which extends beneath the skeleton of the bryozoan host *Celleporaria sibogae* without eliciting a change in the host skeleton [6].

The majority of hydroids are considered to be substrate generalists, living indiscriminately on many different biotic and abiotic surfaces, but in some cases may grow in close association with a living substrate and form specific relationships. Few hydroids live in association with other members of the phylum Cnidaria although they may be common epibionts on the exposed skeletons of dead corals. Those species that do form associations with live Scleractinia [6]–[8] and Octocorallia [5], [9] may live either on the surface of the tissues or embedded within them as partial endosymbionts, with their hydrorhizal system running against the host exoskeleton below the tissues. The net-like hydrorhizal structure ramifies below the host tissues, with individual hydranths of the hydroid colony emerging at the surface through pores in the host tissues [5], [6], [8].

Little is known about the nature of the symbiotic relationships that occur between hydroids and their living hosts. Although there is some evidence of benefits to both host and symbiont in the form of protection from predation and competition for substrate, and direct nutritional source [4], [10]–[13], few studies have looked at the physical interactions between the partners at the cellular level and how these may influence their relationship. Here, we describe the close physical interactions of the recently described perisarc-free capitate hydroid *Zanclea margaritae* that exists as a partial endosymbiont with its scleractinian coral host *Acropora muricata* [8] and explain how this may allow these two colonial animals to co-exist as one without overgrowth by one or other partner.

## Results

### Tissue interactions

**Figure 1:**
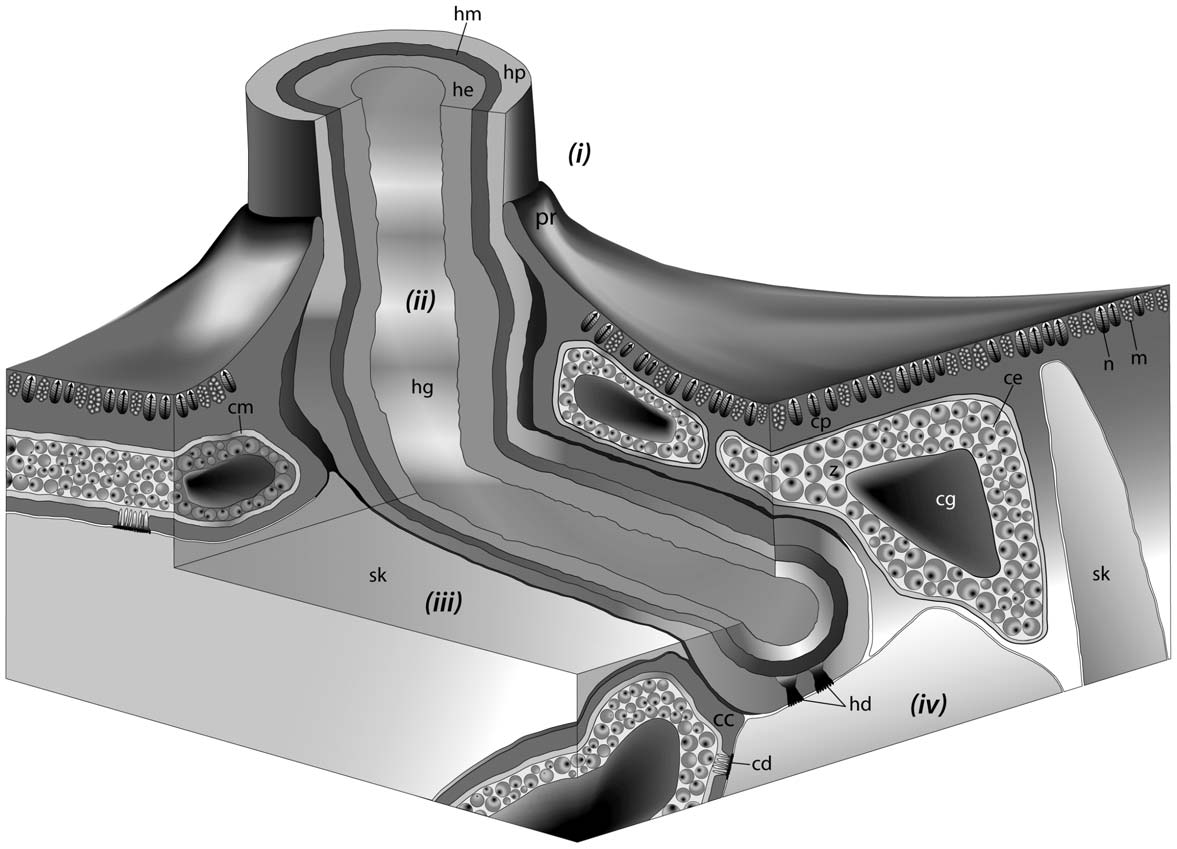
A schematic representation of the physical relationship between the coral host (*Acropora muricata*) and the colonial hydroid (*Zanclea margaritae*). (i) At the surface of the coral colony the hydroid emerges through a pore surrounded by a collar of tissue formed from an extension of the coral epidermis. (ii) The hydroid stolon remains adjacent to the epidermal tissue collar as it passes deeper into the colony. The host epidermal tissue adjacent to the hydroid stolon invaginates and transforms into calicoblastic tissue deeper within the coral, preventing the hydroid from coming in contact with host gastrodermal tissues. (iii) At the position where it first comes into contact with the host skeleton the stolon follows the orientation of the skeletal element. (iv) Both the coral and hydroid employ desmocytes to attach the tissues to the coral skeleton. *pr*, pore rim; *hm*, hydroid mesoglea; *hg*, hydroid gastrovascular cavity; *he*, hydroid endoderm; *hp*, hydroid epiderm; *hd*, hydroid desmocyte; *cp*, coral epiderm; *cm*, coral mesoglea; *cc*, coral calicoblastic tissue; *ce*, coral endoderm; *cg*, coral gastrovascular cavity; *cd*, coral desmocyte; *n*, nematocyst; *m*, mucus cell; *z*, zooxanthellae; and *sk*, skeleton.

**Figure 2:**
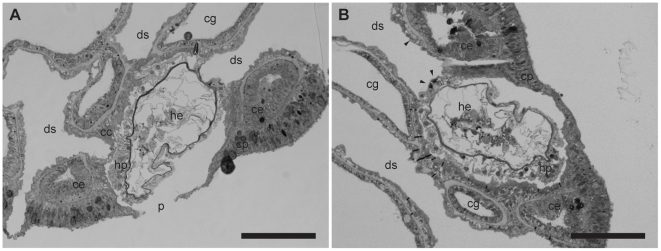
Sequential histological sections showing the location of the partial endosymbiotic hydroid (*Zanclea margaritae*) within the coral host (*Acropora muricata*). (A) The hydroid stolon lies within a cavity of the coral colony extending out to the surface through pores formed from extensions of the epidermal tissue layer. (B) The stolon extends through the coral, away from the stomal opening below the surface tissue layers, and remains in contact with either coral epidermal calicoblastic-like tissues or skeleton. Desmocytes (indicated by arrow heads) are present in both coral and hydroid epidermal tissues that are adjacent to coral skeletal material. Figure labels are described in the legend for Figure 1; *ds*, decalcified skeleton. Scale bars = 100 µm.

**Figure 3:**
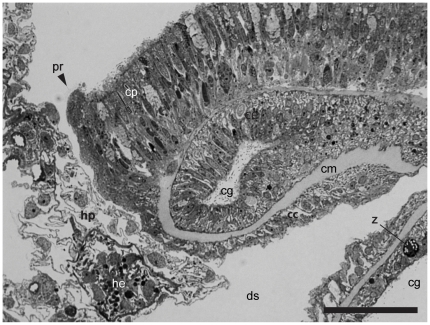
Cross-section of the site of emergence of a hydroid (*Zanclea margaritae*) hydranth through the pore. The surface epidermal layer extends down in to the cavity below the colony surface maintaining contact with the hydroid stolon and forming an inclusion of gastrodermal space. Labelling as in Figure 2. Scale bar = 50 µm.

**Figure 4:**
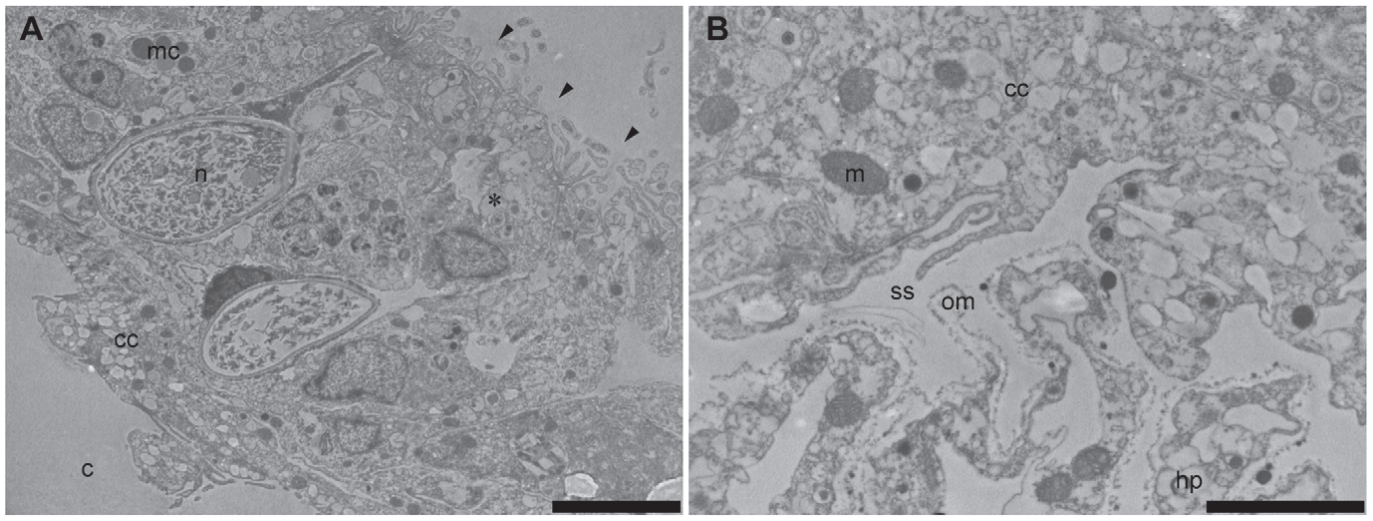
Transmission electron micrographs of the epidermal tissue layer of the surface pore at the coral surface. (A) The single layered epidermal tissue that form the pore possess nematocysts and mucus cells in the upper areas similar to normal surface epidermal tissues (_*_) and the lower area of the tissues are characteristic of calicoblastic tissues, with highly vesiculated elongate cells. (B) The interface between the single-layered epidermal tissues making up the pore and the underlying hydroid tissues. A space may be seen between the tissues of the two organisms, but in some areas the two tissues may appear almost confluent. A layer of organic material lines the hydroid stolon. Adjacent calicoblastic-like coral cell layer are highly vesiculated and have a high density of mitochondria. Arrowheads indicate outer surface of the coral colony. *cc*, coral calicoblast cells; *m*, mitochondria; *c*, cavity; *mc*, mucus cell; *ss*, sub-epithelial space; *om*, organic material; *hp*, hydroid epidermal tissue. Scale bars: A = 5 µm; B = 2 µm.

**Figure 5:**
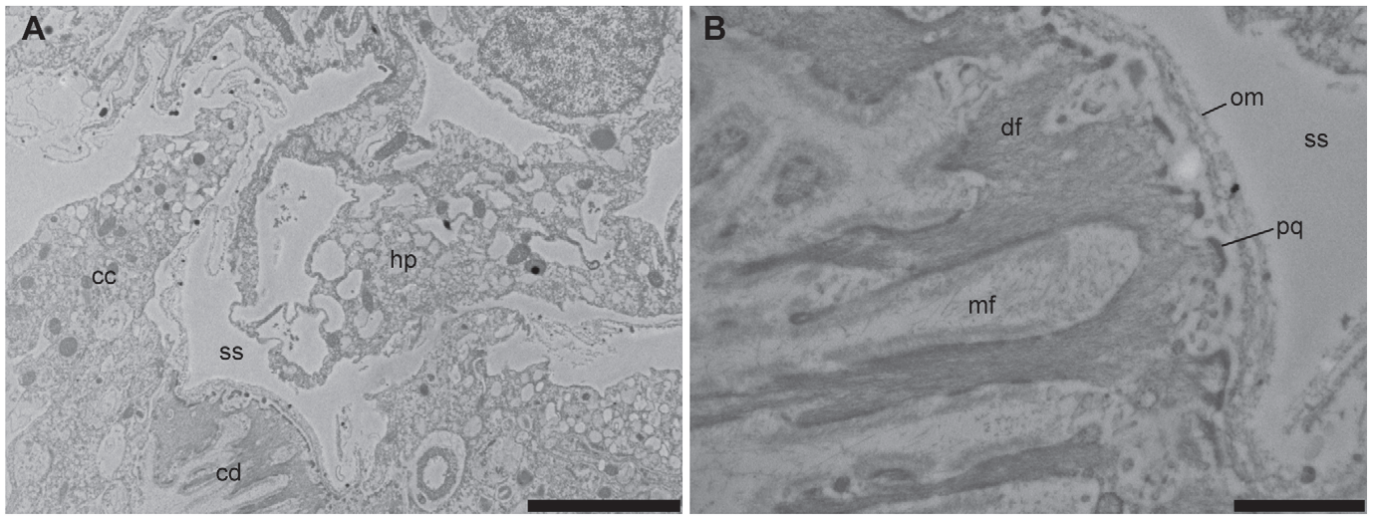
Desmocyte from the tissue of the coral *Acropora muricata* at the site of contact with the endosymbiotic hydroid, *Zanclea margaritae*. (A) Coral desmocytes are present in the calicoblastic tissues facing hydroid tissues suggesting the presence of skeletal material, which may be very thin. (B) Close-up of a coral desmocyte showing the desmocyte tenons extending into the mesoglea, perpendicular to the interface with the skeleton. The matrix of long dense fibres that form the tenons terminate in electron dense plaques. Shorter fibres perpendicular to the tenon rod extend in to the collagen fibre-rich mesoglea. A band of organic material extends across the surface of the desmocyte, in-between the plaques and skeletal material. *cd*, coral desmocyte; *om*, organic material; *hp*, hydroid epidermal tissue; *ss*, sub-epithelial space; *cc*, coral calicoblast; *df*, desmocyte fibres; *mf*, mesogleal fibres; *pq*, plaque. Scale bars: A = 5 µm; B = 1 µm.

**Figure 6:**
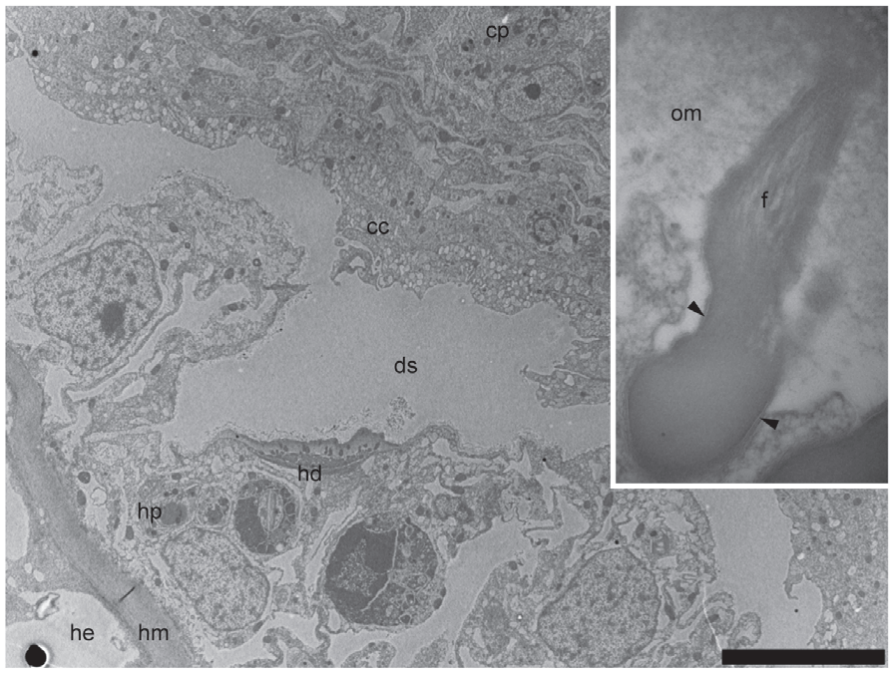
Longitudinal section through the head region of a desmocyte from the hydroid, *Zanclea margaritae*. Mushroom-shaped desmocyte with a broad base and top, and a constricted middle are found in areas of epidermal tissues associated with the coral calicoblastic tissues and skeletal material. Dense accumulation of electron dense filaments form membrane bound tonofibrillar rods (inset, arrowheads indicate membrane) which extend outwards in to the extracellular organic material. *he*, hydroid endoderm; *hm*, hydroid mesoglea; *hp*, hydroid epidermis; *hd*, hydroid desmocyte; *om*, organic material; *cc*, coral calicoblast; *cp*, coral epiderm; *eom*, extracellular organic material; *f*, fibres. Scale bar = 10 µm.

**Figure 7:**
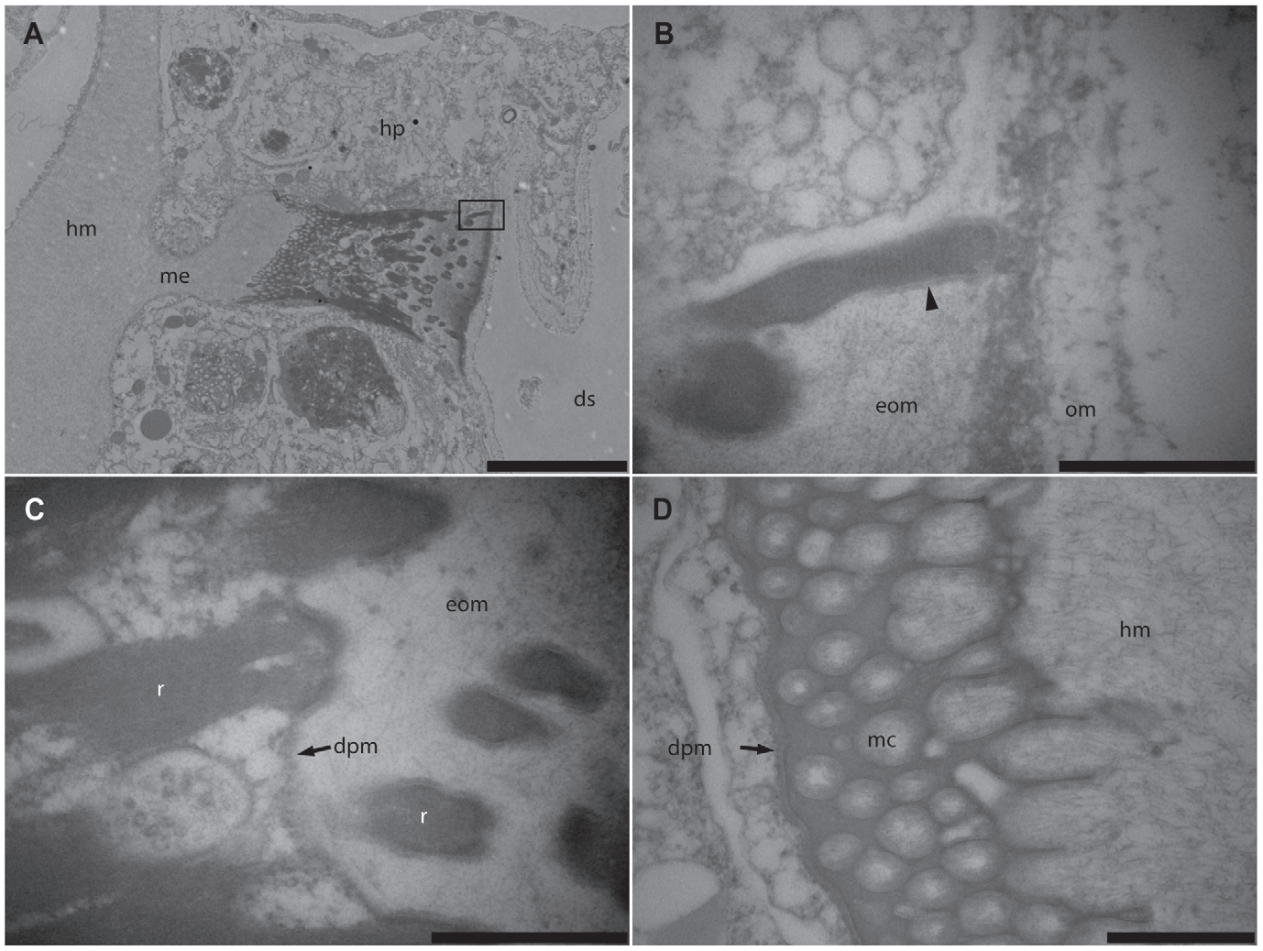
Electron micrographs of a hydroid (*Zanclea margaritae*) desmocyte within the epidermal tissues adjacent to the skeletal material of the coral, *Acropora muricata*. (A) The desmocyte is positioned within the epidermal cell layer, connected to the mesoglea by an extension of the collagenous layer. (B and C) Tonofibrillar rods at the apical end of the desmocytes are transversely striated, perpendicular to the fibres. The rods extend outwards from the apical surface in to an extracellular organic layer adjacent to the carbonate skeleton. (D) Fibrous mesogleal material extends through the interstices of the highly bifurcated distal portion of the desmocyte. *he*, hydroid endoderm; *hm*, hydroid mesoglea; *hp*, hydroid epidermis; *eom*, extracellular organic material; *r*, rod; *dpm*; desmocyte plasma membrane; *mc*, mesogleal channel. Scale bars: A = 5 µm; B–D = 500 nm.

The hydroid hydranths emerge at the surface of the host coral colony surrounded by a collar of host tissues extending outwards (Figures 1 and 2) forming a pore [8]. The collar is composed of an extension of the coral epidermal ectoderm layer, not including either the endodermis or acellular mesoglea (Figure 3). The absence of the endodermal tissue layer at the epithelial pore region which normally contains symbiotic unicellular dinoflagellates giving coral tissues their brown appearance, results in the collar being distinctly paler and near-translucent relative to the surrounding tissue. At the inner rim of the collar adjacent to the hydroid hydranth as it passes through the pore, the epidermal layer thins as it extends down into the coral colony (Figures 1 and 3). As the tissue extends further down into the colony, running parallel to the hydroid stolon, it transforms from epidermal ectoderm to calicoblastic ectoderm. Within the coral colony the hydroid stolon lies adjacent to calicoblastic coral tissues (Figures 1 and 2) and does not pass through gastrodermal space at any point. The ectodermis of the collar is similar in cellular composition to that associated with mesoglea and endodermis in other parts of the coral with high densities of nematocysts and mucocytes close to the surface (Figure 4A). However unlike other areas of endodermis it also has elongate calicoblastic ectodermal cells running parallel to the epithelium surface on the opposite side of the epithelium, adjacent to the hydroid. Deeper within the coral colony, hydroid stolonal tissues are in close association with the calicoblastic layer of the diploblastic epithelial tissues with the two organisms and are separated by less than 1 µm in places (Figure 4B). The calicoblastic tissues are vesiculated, with higher densities of vesicles in tissues associated with areas of skeletal deposition. Mitochondria are very common, but other organelles such as Golgi apparatus and endoplasmic reticulum are rarely seen. Specialised ectodermal cells known as desmocytes, used by certain Cnidaria for the attachment of soft tissues to exoskeletons or hard surfaces, are present within the calicoblastic cells of the coral (Figure 5) and amongst the epidermal tissues of the hydroid (Figures 6 and 7).

Compared to the calicoblastic tissues adjacent to coral skeletal elements, those that face the hydroid stolon were found not to possess intracellular channels which are characteristic of calicoblastic tissues that are not actively laying down skeleton, suggesting that there was no ongoing skeletal extension in this area. However there are some areas of the tissue surrounding the hydroid stolon that have some channels near the surface (Figure 4) from which an extracellular organic material can be seen to be exuded. This exudate is different in appearance to that of the fibrillar organic matrix found adjacent to hydroid coensarc. It appears to be amorphous, may form thin projections, and sometimes also includes organellar debris. The fibrillar organic matrix observed formed a narrow band running parallel to the hydroid's coensarc but does not associate exclusively with either the host or symbiont and was in some cases seen passing from one to the other. In Figure 5A, for example, the band of material is adjacent to the hydroid epidermal layer (top left) and then, moving from left to right, in a continuous layer, becomes associated with the coral tissues. This layer appears to thicken and become more dense when it passes close to the surface of desmocytes.

### Desmocyte Structure

The apical surface of *A. muricata* desmocytes (Figure 5) is round to oval in shape at the tissue-skeleton interface. At the distal end, the desmocytes possess multiple digitate extensions that pass into the mesoglea. The mesoglea protrudes outwards forming an extension into the epidermal tissue layer. These projections, or tenons, were ca. 180 nm in diameter and 3–4 µm long although they may appear shorter depending on where they are sectioned as they taper towards the base and may bifurcate as they extend into the mesoglea. Longitudinal sections through the cells show a pectinate structure with the tenons extending into the mesoglea. Transverse sections reveal that there are multiple rows of tenons, some of which were dendritic. Each tenon was roughly cylindrical with some having irregular cross-sectional profiles (Figure S1) providing a greater surface area for attachment with the mesoglea. The tenons are composed of a matrix of chitinous fibres surrounded by a dense membrane along the lateral boundaries. Shorter fibres positioned perpendicular to the tenons form a fibrillar coat. Theses fibres are shorter and less defined than the long collagen fibres of the mesoglea in which the tenon is embedded. The long dense matrix of fibres within the tenons extends distally and terminates in electron-dense plaques which are linked by the plasma membrane of the desmocytes. When the desmocytes are orientated towards the hydroid stolon, the band of organic matrix that was observed surrounding the hydroid tissues runs close to the desmocyte surface and fibres can be seen traversing the gap.

In contrast to the coral desmocytes which do not differ greatly in width along their length from the distal surface to the proximal, the desmocytes of *Z. margaritae* are mushroom-shaped with a broad, circular top (approximately 10 µm) and base, with a narrowing of the central region (Figures 6 and 7). Dense accumulations of long filaments condense into tonofibrillar rods (average 300 nm at widest point) which are bounded by a defined membrane (Figure 6 inset and 7B) and possess longitudinal ridges giving them a greater surface area. The fibrils are orientated longitudinally within the rods but under high magnification they present a pattern of transverse striations (Figure 7B). The rods protrude from the surface of the cell and become embedded within an extracellular organic matrix, isolated by only the desmocyte plasma membrane (Figure 7B and C). The rods may extend to the surface of this extracellular layer where they abut the organic material identified within the skeletal space. The rods extend towards the base of the cell where they coalesce and the chitinous filaments become distributed within a lattice extension (Figure 7D) which interdigitates with the mesoglea. In contrast to the coral desmocytes the rods are not surrounded by mesoglea but are surrounded by cytoplasmic material (Figure 7A). Similar to the coral desmocytes, the mesoglea is drawn outwards, forming an extension within the epidermal cell layer (Figure 7A). Collagenous mesogleal fibres extend in to the channels formed by the three-dimensional lattice structure of the basal region of the desmocytes, and appear to be concentrated around the parameter of the channels (Figure 7D). At the surface of the extracellular organic matrix in which the desmocyte rods are embedded, fibrils extend towards the band of organic matrix.

### Spatial arrangement of desmocytes

Histological examination by TEM showed that desmocytes were present in the coral and hydroid tissues facing the coral skeleton, including where they border the perimeter of the same skeletal element. Coral desmocytes are located predominately around large skeletal elements, and were frequently found in dense clusters in the epidermal calicoblastic tissue layer but were also found in the tissues that faced the hydroid stolon, separated by less than 1 µm (Figure 4A). Hydroid desmocytes are located within the epidermal layer of the stolon. In areas where the stolon lay adjacent to skeletal elements, multiple desmocytes were found singly or positioned close together in clusters (Figure 2B) forming a larger surface area in contact with the coral skeleton. The desmocytes were also found in the epidermal tissues of the stolon that were in close proximity facing coral calicoblastic tissues (Figure 6).

### Skeletal Microstructure

**Figure 8:**
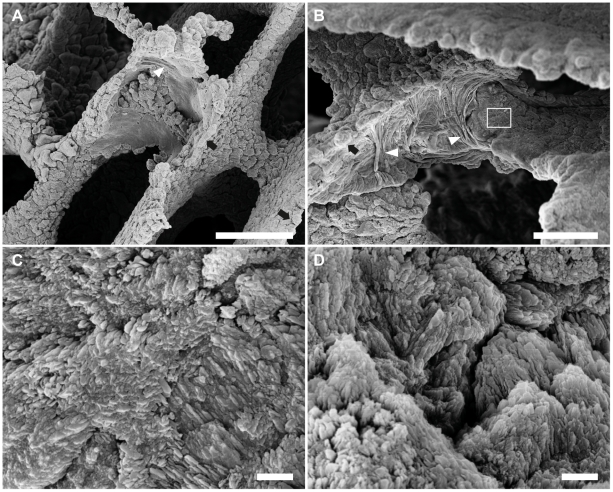
Ultrastructure of the coral skeleton (*Acropora muricata*) is influenced by the symbiotic hydroid, *Zanclea margaritae*. The site of hydranths within the live coral colony corresponds to concave depressions and deformation of the radial sclerosepta. (A) A horizontal element (bar) has a circular depression, and the vertical ridge is flattened with a scooped-out appearance (B). Within the depressions the surfaces appeared smooth, with areas of parallel ridges (arrowheads). Fasciculated nodes lined the edges of the depressions (arrows). (C) Close-up of the boxed area in B. Crystalline surface structure is more finely granulated and does not exhibit the same scale-like clustering found in areas away from hydranths (D). Scale bars: A = 100 µm; B = 50 µm; C and D = 2 µm.

Alterations to the vertical and horizontal skeletal elements of the corallite radial sclerosepta were identified at locations where hydranths had been located prior to removal of the organic material. The sclerosepta appeared unaffected under low magnification but examination by SEM identified changes in the three-dimensional structure and surface texture of both the vertical (rods) and horizontal (bars) elements of the coral skeleton at the sites where hydranths had been located. Concave depressions varied in shape depending on the area of element they were found, with the internal surface being predominantly smooth with areas of smooth-lobed parallel ridges running perpendicular to the vertical elements (Figure 8A and B). Fasciculated nodes lined the perimeter ridge of the depressions, similar to unaffected elements. Where the skeletal surface is crystalline within the depression the crystal arrangement differs to that of the areas away from the zone of depression, having finer granularity and not exhibiting the same scale-like pattern (Figure 8C and D).

## Discussion

Most hydroids are substrate generalists although there are examples from almost all genera that form specific symbiotic relationships with other benthic invertebrates. Some species that form these close relationships are naked having lost their perisarc. This is believed to be a characteristic of symbiosis [2], [4], [10] as the physical support and protection provided by the perisarc is provided by the host skeleton. It is unknown whether these hydroids that are elaborately intertwined with their host colony actively attach to the host or are held in place passively by the surrounding host tissues. Although residing within a host colony offers many benefits it also presents hazards such as antifouling or immune defences and the risk of being overgrown and smothered by the host. How they avoid these detrimental effects but gain the benefits of such an intimate relationship with their living substrates is unclear. In the coral-hydroid system described here, the hydroid was found to attach directly to its host's skeleton, using the same mechanisms employed by its host to attach its own tissues. Desmocytes were found in the epidermal tissues of both coral and hydroid in areas adjacent to the skeleton. Desmocytes are found within the epidermal tissue layers of various cnidarian taxa and attach soft tissues to the mineral and chitinous exoskeletons [14]–[21], or where an exoskeleton is absent such as in soft corals and the polyp stages of the Medusozoa, anchor the tissues to hard benthic substrata [15], [22], [23]. In corals, desmocytes facilitate the attachment and subsequent release of the tissues to the skeleton as it is accreted by the calicoblastic layer [14], [16], [19], allowing the tissues to maintain their relative position on the colony; although the exact mechanisms involved are still unknown. As they are not directly associated with the accretion of skeletal material in corals they are not present at higher densities in actively calcifying zones, but are more common in areas where morphological development is complete or where mechanical forces are exerted such as at the site of mesentery insertions [14], [19], [20], [24]. Desmocytes mediate the support of the soft coensarc by linking the acellular mesogleal tissue layer to the rigid chitinous tube in hydroids that possess a perisarc, preventing slippage whilst still allowing movement of the hydranths for feeding, defence and predator avoidance [15], [18], [21], [25]. New desmocytes differentiate from the epidermal cells of the stolon and attach to the perisarc as the stolon extends, with older redundant desmocytes becoming detached which gradually degrade. This results in attachment occurring only within the apical zone of the chitinous tube with desmocytes occurring singly and at random intervals [18]. The lack of either complete or partial encasing of the hydroid due to bioclaustration, which is found to occur around other hydroids that live within live hosts [5], [10], [26], means that attachment around the whole stolon is not possible and is potentially reduced. In the coral-hydroid association described here, the point of attachment occurs within the cup-shaped depressions of the skeleton that were associated with hydranths. Although this area of attachment may be limited, strong attachment appears to be achieved through the properties associated with desmocytes. Differences in desmocyte structure are consistent with other observations of members of the Anthozoa and Medusozoa [15], [16], [18], [19], [21], [22], [24], [25], [27]. The high level of interdigitation at the distal end of the hydroid desmocytes is indicative of the strong anchorage within the mesoglea, and the large surface area of the apical end of individual desmocytes suggests a strong attachment to the coral skeleton. Desmocytes within these areas were also found clustered together, an arrangement similar to that found in coral desmocytes at sites of high physical stress [14], [19], [20], [27]. The extracellular organic material present at the apical surface of the hydroid desmocytes into which the tenons extend is also similar to that seen in the polyp stages of soft corals, jellyfish and hard corals for the attachment to hard substrata or calcium carbonate skeletons. This material acts as a glue between the desmocytes and the substrate, forming a strong bond and anchorage for the tissues to the substrate or skeleton [19], [22], [23].

The occurrence of discrete attachment points on the coral skeleton characterised by the concave area of skeleton with altered surface properties suggests that the pattern of attachment is similar to that of the attachment of a stolon within its perisarc. Desmocytes occur just below the apical end of the perisarc forming a discrete area of attachment, attaching the stolon to the chitinous tube. Below this point of attachment the stolon is not attached to the perisarc and does not remain in close contact [18]. The smoothing of the coral skeleton surface within these areas of hydranth suggests that this is the only area in which the hydroid becomes attached to the skeleton, affecting its accretion and consequently its physical properties [20], [28]. The hydrorhiza, which has been found to extend deep within the skeleton [8], may extend from this point running beneath the tissues without attaching to the skeleton, thereby not affecting its formation and resulting in the observed single-point attachment scars.

It is unclear whether the progressive detachment and reattachment of new desmocytes similar to hydroids with a perisarc [18] occurs in *Z. margaritae* colonies growing within the coral host. Such a progression would allow them to maintain their relative position with the host tissues. Nor is it known whether modulated adhesion occurs, as is thought to occur in corals [14], [16], [19] where the desmocytes detach during periods of secretion of skeletal mineral followed by re-attachment to newly secreted surfaces. Either mechanism would allow the hydroid to avoid being overgrown by the host skeleton, and eventually being encapsulated and killed. The necessity to be able to maintain their relative position and the period of time required may be dictated by their lifecycle and influenced by the growth rate of the coral. As the length of the different life stages or details of the full life cycle remain unknown for this species it is not possible to say whether they would need to be able to keep up with this fast growing coral species to complete the polyp stage of their life cycle or whether the colony persists within the coral after the release of the medusae [8]. Differences in both the surface structure and shape of the skeletal elements at the sites of the hydranths suggest, however, that these elements may remain in the same position for an extended period of time.

The success of benthic organisms is strongly influenced by competition for space. Sessile benthic organisms utilise a range of antifouling mechanisms to avoid competition after settlement. The ability to circumvent these strategies and successfully settle on and grow within living organisms allows hydroids to be highly competitive for space by exploiting otherwise inaccessible substrates and in gaining access to new ecological niches. Although living within a colonial host provides many benefits, it presents a series of challenges. One of these is associated with growing within a colonial organisms and maintaining a relative position within the colony and avoiding being smothered as a consequence of host growth. The loss of perisarc is thought to have occurred over time where the host colony provides physical support and protection, therefore rendering it redundant. However, its absence enables *Zanclea margaritae* to attach to the host skeleton using a strong but dynamic attachment which is unlike the glue-like permanent attachment used for the adhesion of perisarc to the substrate. During periods of skeletal extension, the stolon may be released similar to the process of perisarc elongation, enabling the hydroid to maintain its relative position within the colony. This ability to avoid overgrowth by host skeleton and therefore maintain their position within their host is likely to be fundamental to the success of *Z. margaritae* in exploiting the skeleton of a member of the fast growing staghorn corals [29]–[32].

This is the first study to show that hydroids that have lost their perisarc and live endosymbiotically with a host species have retained their desmocytes; and use them to provide a potentially dynamic attachment to their host's skeleton to gain physical support. Other similar naked hydroids living within a colonial host may also have retained their desmocytes enabling them to exploit valuable settlement space and circumvent some of the challenges that these heavily defended dynamic landscapes present that other species are not able to access.

## Materials and Methods

Branch tips were collected from colonies of *Acropora muricata* from Heron Island Reef, Great Barrier Reef, Australia (23°26′31.20″S 151°54′50.40″E) under Great Barrier Reef Marine Park Authority collection permit G07/23038.1. No ethical approval was required for the experimental research described here. Nubbins were transported to aquaria in sealed containers avoiding exposure to air. Individual coral corallites possessing emergent hydranths of *Zanclea margaritae* were excised from the branch tip whilst submerged in seawater using a mounted razor blade and transferred directly to 2% glutaraldehyde in artificial seawater using a wide-bore Pasteur pipette avoiding physical handling of the tissues. Further fixation and embedding was carried out using a microwave-assisted method in 2% glutaraldehyde in 0.1 M cacodylate buffer and samples were post-fixed in 1% osmium tetroxide in cacodylate buffer. Tissues were dehydrated through an ascending ethanol series and embedded in LR White histological resin. Ultrathin sections (ca. 65 nm) were prepared using a diamond knife on a Leica Ultracut UCT (type 706201) ultramicrotome and mounted on Formvar coated copper slot grids. Sections were stained with saturated uranyl acetate in 50% ethanol and counterstained with lead citrate before examination using a JEOL JEM-1010 transmission electron microscope with a beam energy of 80 kV. For examination of the coral skeletal surface by scanning electron microscopy (SEM), the tissue and organic matter was removed from individual *A. muricata* polyps using a dilute sodium hypochlorite solution. Individual coral calices were mounted on appropriate stubs using self-adhesive carbon conductive tabs and then sputter-coated with gold. Before removal of the tissues the location of individual hydranths were identified on each calix. The skeletons were then viewed with a JEOL NeoScope Benchtop SEM operating at beam energies of 10 and 15 kV.

## Supporting Information

Figure S1 **Transmission electron micrograph of an oblique plane section of a coral desmocyte close to the site of the endosymbiotic hydroid,** ***Zanclea margaritae*** **.** Roughly cylindrical tenons (indicated by arrow head) extend in to the fibrillar mesoglea. Their irregular cross-sectional profiles provide a greater surface area for attachment within the mesoglea. *df*, desmocyte fibres; *mf*, mesogleal fibres; *pq*, plaque. Scale bar = 2 µm.
(TIF)

## References

[1] Millard NAH (1975). Monograph on the Hydroida of southern Africa.. Ann S Afr Mus.

[2] Bouillon J, Gravili C, Pagès F, Gili JM, Boero F (2006). An introduction to Hydrozoa.

[3] Puce S, Bavestrello G, Di Camillo CG, Boero F (2007). Symbiotic relationships between hydroids and bryozoans.. Symbiosis.

[4] Ristedt H, Schuhmacher H (1985). The bryozoan *Rhynchozoon larreyi* (Audouin, 1826): A successful competitor in coral reef communities of the Red Sea.. Mar Ecol.

[5] Puce S, Di Camillo CG, Bavestrello G (2008). Hydroids symbiotic with octocorals from the Sulawesi Sea, Indonesia.. J Mar Biol Assoc UK.

[6] Boero F, Bouillon J, Gravili C (2000). A survey of *Zanclea*, *Halocoryne* and *Zanclella* (Cnidaria, Hydrozoa, Anthomedusae, Zancleidae) with description of new species.. Ital J Zool.

[7] Millard NAH, Bouillon J (1973). Hydroids from the Seychelles (Coelentara).. Ann Mus Roy Afr Cen.

[8] Pantos O, Bythell JC (2010). A novel reef coral symbiosis.. Coral Reefs.

[9] Gili JM, Lopez-Gonzalez PJ, Bouillon J (2006). A new Antarctic association: the case of the hydroid *Sarsia medelae* (new sp.) associated with gorgonians.. Polar Biol.

[10] Osman RW, Haugsness JA (1981). Mutualism among sessile invertebrates - A mediator of competition and predation.. Science.

[11] Piraino S, Bouillon J, Boero F (1992). *Halocoryne epizoica* (Cnidaria, Hydrozoa), a hydroid that “bites”.. Scientia Marina.

[12] Piraino S, Todaro C, Geraci S, Boero F (1994). Ecology of the bivalve-inhabiting hydroid *Eugymnanthea inquilina* in the coastal sounds of Taranto (Ionian Sea, SE Italy).. Mar Biol.

[13] Rees WJ (1967). A brief survey of the symbiotic associations of Cnidaria with Mollusca.. Malacol Soc London Proc.

[14] Brahmi C, Meibom A, Smith DC, Stolarski J, Auzoux-Bordenave S (2010). Skeletal growth, ultrastructure and composition of the azooxanthellate scleractinian coral *Balanophyllia regia*.. Coral Reefs.

[15] Chapman DM (1969). The nature of cnidarian desmocytes.. Tissue and Cell.

[16] Goldberg WM (2001). Desmocytes in the calicoblastic epithelium of the stony coral *Mycetophyllia reesi* and their attachment to the skeleton.. Tissue Cell.

[17] Kingsley RJ, Watabe N (1987). Role of carbonic-anhydrase in calcification in the gorgonian *Leptogorgonia virgulata*.. Journal of Experimental Zoology.

[18] Marcum BA, Diehl FA (1978). Anchoring cells (Desmocytes) in hydrozoan polyp *Cordylophora*.. Tissue Cell.

[19] Muscatine L, Tambutte E, Allemand D (1997). Morphology of coral desmocytes, cells that anchor the calicoblastic epithelium to the skeleton.. Coral Reefs.

[20] Tambutte E, Allemand D, Zoccola D, Meibom A, Lotto S (2007). Observations of the tissue-skeleton interface in the scleractinian coral *Stylophora pistillata*.. Coral Reefs.

[21] Tidball JG (1982). Fine-structural aspects of anthozoan desmocyte development (Phylum Cnidaria).. Tissue Cell.

[22] Barneah O, Malik Z, Benayahu Y (2002). Attachment to the substrate by soft coral fragments: desmocyte development, structure, and function.. Invert Biol.

[23] Hofmann DK, Honegger TG (1990). Bud formation and metamorphosis in *Cassiopea andromeda* (Cnidaria, Scyphozoa) - A developmental and ultrastructure study.. Mar Biol.

[24] Bourne GC (1899). Studies on the structure and formation of the calcareous skeleton of the Anthozoa.. Quart J Micr Sci.

[25] Bouillon J, Levi C (1971). Structure and ultrastructure of anchoring devices of hydrants in Thecata.. Zeitschrift Fur Zellforschung Und Mikroskopische Anatomie.

[26] McKinney F (2009). Bryozoan-Hydroid symbiosis and a new Ichnogenus, *Caupokeras*.. Ichnos-an International Journal for Plant and Animal Traces.

[27] Goldberg WM (2001). Acid polysaccharides in the skeletal matrix and calicoblastic epithelium of the stony coral *Mycetophyllia reesi*.. Tissue Cell.

[28] Abramovitch-Gottlib L, Dahan D, Golan Y, Vago R (2005). Effect of light regimes on the microstructure of the reef-building coral Fungia simplex.. Materials Science & Engineering C-Biomimetic and Supramolecular Systems.

[29] Barnes DJ, Crossland CJ (1980). Diurnal and seasonal variations in the growth of a staghorn coral measured by time-lapse photography.. Limnol Oceanogra.

[30] Gladfelter EH, Monahan RK, Gladfelter WB (1978). Growth rates of five reef-building corals in the northeastern Caribbean.. Bull Mar Sci.

[31] Lirman D (2000). Fragmentation in the branching coral *Acropora palmata* (Lamarck): growth, survivorship, and reproduction of colonies and fragments.. J Exp Mar Biol Ecol.

[32] Shinn EA (1966). Coral growth-rate, an environmental indicator.. J Paleontol.

