# Supplementary figures and images for: Shared Skeletal Support in a Coral-Hydroid Symbiosis
Source: PLoS One. 2011 Jun 14;6(6):e20946. doi: 10.1371/journal.pone.0020946 (PMC3114865; doi:10.1371/journal.pone.0020946)

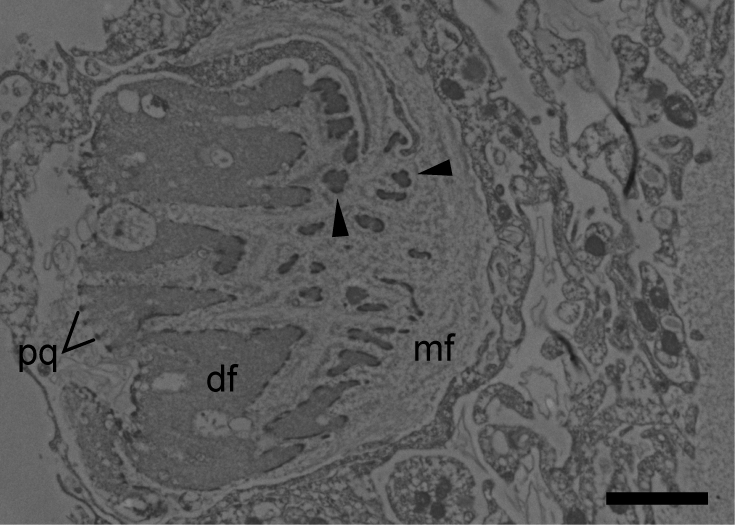

Supplement: Figure S1 — Transmission electron micrograph of an oblique plane section of a coral desmocyte close to the site of the endosymbiotic hydroid, Zanclea margaritae . Roughly cylindrical tenons (indicated by arrow head) extend in to the fibrillar mesoglea. Their irregular cross-sectional profiles provide a greater surface area for attachment within the mesoglea. df, desmocyte fibres; mf, mesogleal fibres; pq, plaque. Scale bar = 2 µm. (TIF) [file pone.0020946.s001.tif]
